# Partial Ablation of Postsynaptic Dopamine D2 Receptors in the Central Nucleus of the Amygdala Increases Risk Avoidance in Exploratory Tasks

**DOI:** 10.1523/ENEURO.0528-21.2022

**Published:** 2022-03-15

**Authors:** Eric Casey, María Elena Avale, Alexxai Kravitz, Marcelo Rubinstein

**Affiliations:** 1 Instituto de Investigaciones en Ingeniería Genética y Biología Molecular, Consejo Nacional de Investigaciones Científicas y Técnicas, Buenos Aires 1428, Argentina; 2 Departamento de Fisiología, Biología Molecular y Celular, Facultad de Ciencias Exactas y Naturales, Universidad de Buenos Aires, Buenos Aires 1428, Argentina; 3 Department of Anesthesiology, Washington University St. Louis, St. Louis, MO 63108; 4 Department of Psychiatry, Washington University St. Louis, St. Louis, MO 63108; 5 Department of Neuroscience and Biomedical Engineering, Washington University St. Louis, St. Louis, MO 63108

## Abstract

The central nucleus of the amygdala (CeA) is involved in the expression of fear and has been implicated in several anxiety disorders. This structure is densely innervated by DAergic projections that impinge on amygdalar neurons expressing various dopamine (DA) receptor subtypes, including D2 receptors (D2Rs). Although various pharmacological approaches have assessed the role of D2Rs in the CeA, the actual participation of postsynaptic D2Rs in the CeA to defensive behaviors remains unclear. Here, we investigated the distribution of D2Rs in the CeA and their role in modifying neuronal activity and fear related behaviors in mice. First, using the mouse reporter strain D2R-EGFP, we verified that D2Rs are present both in neurons of the CeA and in A10 dorsocaudal (A10dc) DAergic neurons that innervate the CeA. Moreover, we showed that pharmacological stimulation of D2Rs increases the activity of protein kinase C (PKC)δ cells present in the CeA, a type of neuron previously associated with reduced defensive behaviors. Finally, using a molecular genetics approach that discriminates postsynaptic D2Rs from presynaptic D2 autoreceptors, we demonstrated that mice carrying targeted deletions of postsynaptic D2Rs in the CeA display increased risk avoidance in exploratory tasks. Together, our results indicate that postsynaptic D2Rs in the CeA attenuate behavioral reactions to potential environmental threats.

## Significance Statement

The central nucleus of the amygdala (CeA) is a neural hub involved in risk assessment and fear-related behaviors, and its malfunction may trigger anxiety disorders. The CeA is densely innervated by dopamine (DA) projections that activate D1 receptors (D1Rs) and D2Rs. In this study, we sought to determine the role that postsynaptic D2Rs in the CeA exert in defensive behaviors. We first showed that pharmacological stimulation of D2Rs increases the activity of neurons known to reduce defensive behaviors. We also showed that mice partially lacking postsynaptic D2Rs in the CeA display increased risk avoidance in exploratory tasks. Together, our results indicate that D2Rs in the CeA attenuate behavioral reactions to potentially aversive environmental stimuli rising new perspectives to manage anxiety disorders.

## Introduction

Risk assessment, fear, and threat avoidance are highly conserved adaptive behaviors that are essential for fitness and survival. In some cases, however, fear responses appear as exaggerated behavioral reactions to stimuli that do not represent a commensurate threat, underling symptoms of pathologic conditions such as anxiety and posttraumatic disorders. Uncontrollable anxiety may also lead to compulsive behaviors commonly observed during withdrawal from major abused drugs such as opioids, cocaine, nicotine, and ethanol (Koob, 2008).

The central nucleus of the amygdala (CeA) is a neural hub that orchestrates innate and learned fear-related behaviors. The CeA is densely innervated by DAergic fibers arriving mainly from A10 dorsocaudal (A10dc) neurons located in the ventral periaqueductal gray (vPAG) and dorsal raphe (DR; Hasue and Shammah-Lagnado, 2002; Li et al., 2016). The dopamine (DA) D2 receptor gene (*DRD2*) is expressed in the CeA of rodents (Scibilia et al., 1992; Kim et al., 2017; McCullough et al., 2018a,b) and humans (Gurevich and Joyce, 1999; Xiang et al., 2008). In mice, DA D2 receptors (D2Rs) present in the CeA have been implicated in impulsive behaviors (Kim et al., 2018), whereas several association human studies have linked particular genetic polymorphisms of *DRD2* with avoidance behavior (Frank and Hutchison, 2009), social phobia (Schneier et al., 2000), social dysfunction (Lawford et al., 2006), and anxiety-driven alcoholism (Joe et al., 2008). Given the involvement of the CeA in defensive behaviors, and its regulation by DA, we hypothesized that D2Rs in the CeA regulate behavioral responses to potential threats, and that ablation of postsynaptic D2Rs in the mouse CeA would increase avoidance behaviors. Although previous studies have used local applications of antagonists to evaluate the function of D2Rs in the CeA (Guarraci et al., 2000; De la Mora et al., 2012; De Bundel et al., 2016), pharmacological approaches are unable to discriminate between the blockade of presynaptic and postsynaptic D2Rs.

In this study, we sought to investigate the role of amygdalar D2Rs in defensive and fear related behaviors. To this end, we first evaluated the distribution of postsynaptic and presynaptic D2Rs in the CeA. Then, we determined the pattern of CeA neurons activated by pharmacological stimulation of D2Rs. Finally, we used a molecular genetics approach to partially eliminate postsynaptic D2Rs from the CeA and studied their risk assessment behaviors in approach/avoidance conflict paradigms. Altogether, our results support the hypothesis that postsynaptic D2Rs in the CeA play an active role in threat assessment of environmental cues.

## Materials and Methods

### Mice husbandry

Mice of both sexes were housed in ventilated cages under controlled temperature and photoperiod (12/12 h light/dark cycle, lights on from 7 A.M. to 7 P.M.), with tap water and laboratory chow available *ad libitum,* and separated by sex. For behavioral experiments and drug administration 7- to 16-week-old mice were transferred to an experimental animal room with similar housing conditions and allowed for at least one week of habituation before experiments. All procedures followed the *Guide for the Care and Use of Laboratory Animals*, United States Public Health Services (2011) and in agreement with the INGEBI-CONICET Institutional Animal Care and Use Committee. *Drd2*−/− and *Drd2^loxP/loxP^* mice were generated by crossing a heterozygote male mouse carrying the original floxed exon 2- floxed PGK-neo *Drd2* allele with a B6.FVB-Tg(EIIa-cre)C5379Lmgd/J female (The Jackson Laboratory) and then backcrossed for >10 generations to C57BL/6J, as previously described in detail (Bello et al., 2011). *Drd2^loxP/loxP^* mice are conditional mutants carrying targeted loxP sites flanking *Drd2* exon 2 (*Drd2^tm1.1Mrub^*/J). *Drd2^loxP/loxP^*, *Drd2*−/−, *Dat^+/IRES-Cre^* (Bäckman et al., 2006), *Drd2*-EGFP (Gong et al., 2003), and Ai14 (Madisen et al., 2010) mice were all bred in our facility and maintained in a C57BL/6J background.

### Stereotaxic surgeries

Mice were anesthetized with ketamine (100 mg/kg, i.p.) and xylazine hydrochloride (10 mg/kg, i.p.). A 10-μl Hamilton syringe connected with a 36-gauge metal needle was used to infuse lentiviral vectors using a microsyringe pump at 0.1 μl/min. Stereotaxic coordinates for the CeA were in relation to the Bregma (Paxinos and Franklin, 2008): anterior-posterior, −1.5 mm; medial-lateral, ±3.0 mm; dorsal-ventral, −4.9 mm. Following infusion, the needle was kept at the injection site for 5 min, and then slowly withdrawn to half way, kept there for two more minutes and then slowly withdrawn outside the brain. Skin was sutured, local anesthesia (lidocaine gel) was applied followed by the analgesic flunixin meglumine (5 mg/kg, s.c.). Mice were maintained on a regulated warm pad and monitored until recovery from anesthesia.

Mice received 0.6 μl of a solution containing 3.3 × 10^8^ particles/ml directly into the CeA, bilaterally. CeA*Drd2*KO mice were generated by stereotaxic co-injections of LV:GAD-Cre (1.65 × 10^8^ viral particles/ml) and LV:Ub-EGFP (1.65 × 10^8^ viral particles/ml) into the CeA of *Drd2^loxP/loxP^* mice whereas control mice received injections of LV:Ub-EGFP (3.3 × 10^8^ particles/ml) alone. The same procedure was performed for LV:GAD-Cre and LV:Ub-EGFP injections in *wild-type* mice (*Drd2*^+/+^) for control experiments.

### Lentiviral preparations

HEK-293T cells were grown on high glucose DMEM (Invitrogen), supplemented with 10% (v/v) fetal bovine serum (Natocor), 0.5 mm L-glutamine, 100 U/ml penicillin and 100 μg/ml streptomycin. Cells at 80–85% confluence in 100-mm plates were co-transfected with 3 μg of the lentiviral shuttle vector (either GAD-CRE or Ub-EGFP) together with helper vectors encoding packaging and envelope proteins (CMVΔ8.9 and CMV-VSVg, 3 and 1.5 μg, respectively), using lipofectamine (Plus reagent, Thermo Fisher Scientific). Viral particles were harvested from the culture medium 36 h after transfection, treated with RNase-free DNase I (Invitrogen), filtered and concentrated by ultracentrifugation at 100,000 × *g* (Ti 90 rotor, Beckman), yielding viral suspensions at a titer of 10^7^ TU/ml. LV aliquots were stored at −80°C and thawed on ice before use. LV:GAD-Cre carries Cre recombinase coding sequences driven by the mouse GAD67 promoter whereas LV:Ub-EGFP carries EGFP coding sequences driven by the human ubiquitin promoter. Both viral vectors contain a woodchuck hepatitis virus posttranscriptional regulatory element (WPRE), long terminal repeats (LTR), RNA pack and genomic RNA packaging signals, a rev response element (RRE), a central polypurine tract (cPPT), a central termination sequence (CTS), a 3′ end PPT (3-PPT), and a ΔU3 400-bp deletion in the 3′ LTR.

### Tissue collection and histology

Transcardiac perfusions were performed with PBS (0.9% NaCl, 2.7 mm KCl, 10 mm K_2_HPO_4_, and 2 mm KH_2_PO_4_, pH 7.5) followed by paraformaldehyde 4% in PBS and brains were removed and postfixed in the same solution at 4°C for 12–16 h. Brains were sectioned at 40 μm on a vibratome (Leica) and used immediately or stored at −20°C in a solution containing 30% (v/v) ethylene glycol, 30% (v/v) glycerol and PBS, until they were processed for immunofluorescence. Immunolabeling was performed as follows: free-floating sections were rinsed three times for 10 min in PBS. For protein kinase C (PKC)δ immunofluorescence an antigen-retrieval protocol was applied: following PBS rinse, sections were incubated in citrate buffer (10 mm citric acid and 0.05% Tween 20, pH 6.0) at 95°C for 5 min and then rinsed three times for 10 min in PBS. Sections were incubated for 16 h at 4°C in primary antibody solution with normal goat serum 2% (w/v), 0.3% Triton X-100, in PBS. The following primary antibodies were used: rabbit anti-TH (1:2000; Millipore, AB5935), chicken anti-TH (1:1000; Abcam, AB76442), rabbit anti-C-FOS (1:500, Santa Cruz Biotechnology, SC-52), mouse anti-PKCδ (1:500, BD Biosciences, 610398), and chicken anti-EGFP (1:1000; Aves, GFP-1020). After incubation with a primary antibody, sections were rinsed twice for 20 min in PBS and then incubated for 2 h at room temperature with goat or donkey Alexa Fluor 488- or Alexa Fluor 555-coupled secondary antibody 1:1000 in 0.3% Triton X-100 in PBS. Finally, sections were rinsed twice for 20 min in PBS and mounted with Vectashield (Vector Labs) for confocal microscopy or glycerol 50% (v/v) in PBS for fluorescence and bright field microscopy.

### Microscopy and images analysis

Confocal images for coexpression assays and quantification were obtained using a Leica Confocal TCS-SPE microscope. Images not used for colocalization analysis were obtained by fluorescence microscopy. Images were analyzed with the Fiji platform (Schindelin et al., 2012) of the ImageJ software (Rueden et al., 2017). For c-FOS quantification, cell number was semi-automatically quantified with the tool “Analyze Particles” after manually delimitating the region of interest. Colocalization images were obtained by confocal microscopy and was manually quantified using the tool “Cell Counter.”

### Experimental design and statistical analyses

#### Drug administration and c-FOS detection

Mice of both sexes older than eight weeks were used. All drugs were dissolved in NaCl 0.9% to reach a concentration such that the injected volume was 0.1 ml per 10 g of body weight. Experiments evaluating cocaine and quinpirole effects were performed separately, and because of that, they were not analyzed in the same statistical analysis. Vehicle (NaCl 0.9%), cocaine hydrochloride (20 mg/kg; Sigma) or quinpirole (1 mg/kg; Sigma) were injected intraperitoneally. This dose of quinpirole was selected based on a previous report showing increased expression of the neuronal activation marker P-rpS6 in CeA neurons (De Bundel et al., 2016). Mice were left in their home cages and 90 min later were perfused for tissue fixation and histology.

The number of c-FOS immunostained cells per hemisphere and coronal section (between two and five per mouse) was obtained. For plotting, the average number of cells between hemispheres and sections of the same mouse was calculated. For statistical analysis, sections corresponding to the same mouse were treated as subsamples of the same mouse. Colocalization of c-FOS and PKCδ was analyzed with a two-way generalized linear mixed model (GLMM) with Poisson distribution or negative binomial distribution, with Drug as between-subjects factor with two levels (vehicle and quinpirole, or vehicle and cocaine), Neuronal population as intrasubjects factor with two levels (PKCδ+ and PKCδ–), Mouse as random variable, and Section-hemisphere as subsamples (Section-hemisphere nested in Mouse). c-FOS in *wild-type* versus *Drd2*KO mice was analyzed with a two-way GLMM with negative binomial distribution, with Drug as between-subjects factor with two levels (vehicle and quinpirole), Genotype as between-subjects factor with two levels (*wild-type* and *Drd2*KO), Mouse as random variable and Section-hemisphere as subsamples (Section-hemisphere nested in Mouse). Significance was evaluated with the likelihood-ratio test (LRT). *Post hoc* Tuckey’s multiple comparisons were performed.

#### Behavioral tests

*Drd2^loxP/loxP^* male mice older than eight weeks were injected with a combination of LV:GAD-Cre and LV:Ub-EGFP (CeA*Drd2*KO group) or LV:Ub-EGFP alone (control group), and after two to three weeks were evaluated in a battery of tests to evaluate avoidance behaviors [open field (OF), dark/light box test (DLBT), and elevated plus maze (EPM)] and fear conditioning (FC). As a control experiment, *wild-type* male mice older than eight weeks received identical surgeries and behavioral tests except for the FC (LV:GAD-Cre group and LV:Ub-EGFP group). Two littermate cohorts were used to reach sample size per group (CeA*Drd2*KO = 9, Ctrl = 8; GAD-Cre = 7, Ub-EGFP = 7).

Differences in avoidance behavior were evaluated with a multivariate ANOVA (MANOVA) including the values obtained from the OF (time in center), the DLBT (time in light, latency to first enter to light and number of entries to light), and the EPM [percentage of time on open arms or normalized time on open arms (see below, EPM), and entries to open arms]. In addition, the results of the MANOVA were confirmed with individual univariate tests (described in the section of each test).

##### OF exploration test

Horizontal locomotion and exploration in a novel OF were evaluated in activity boxes (Med Associates) for 30 min for three consecutive days. Total distance traveled during the first 5 min of each day and the time in the center of the arena were determined using the software Activity Monitor (Med Associates). Time in center was evaluated by *t* test. Distance was evaluated in a two-way ANOVA with repeated measures, with group as between-subjects factor and day as intrasubject factor with three levels (day 1, day 2, and day 3).

##### EPM

We used a custom-made apparatus standing 50 cm above the floor and constructed with black acrylic. Each arm of the maze is 5 cm wide and 30 cm long. The closed arms have black acrylic walls, 12 cm high. Mice were individually placed in the center of the maze and allowed to explore for 5 min while being videotaped. Entry to an arm was counted when more than half of the body of the mouse was inside the arm. Percentage of time on open arms was calculated with the formula: 100 × time on open/(time on open + time on closed); percentage of entries to open was calculated with the formula: 100 × entries to open/(entries to open + entries to closed). Univariate differences in percentage of time spent on the open arms were evaluated with a *t* test; univariate differences in percentage of entries to open arms were evaluated with Wald test in a generalized linear model (GLM) with quasi-binomial distribution. Only for the percentage of time spent on the open arms in *Drd2^loxP/loxP^* mice, significant effect of the cohort was detected. In addition, the variance also differed between cohorts, causing a non-normal distribution of the residuals. Therefore, the data were analyzed in two different ways: first, using a two-way ANOVA with group and cohort as between-subjects’ factors with variance modeling (VarIdent function, library “nlme”); second, dividing each value by the average of its respective cohort (independently of the group) and analyzing this normalized data with a *t* test. Since the normalization restored the normal distribution of the residuals, normalized data were used for the MANOVA of *Drd2^loxP/loxP^* mice instead of the original percentage of time spent on the open arms.

##### Light/dark box test

A custom-made two-chamber shuttle box containing a dividing wall with a 4 × 5 cm hole in the center that allows mice free access to both sides was used. Each chamber is 20 (w) × 26 (l) × 14 cm (h) with steel walls and floor. Mice were placed on one side of the shuttle box that was then immediately covered with a black acrylic ceiling. The other chamber received ambient illumination. Mice were allowed to explore the chambers for 5 min while videotaped. The time before entering into the lit compartment (latency), the time spent on the illuminated side, and the number of entries to the lit compartment were determined. Entry to or exit from the lit compartment was counted when the mouse passed more than half of its body. Univariate differences in latency and time spent on the illuminated chamber were evaluated by *t* test. Univariate differences in number of entries were evaluated with Wald test in a GLM with Poisson (*Drd2^loxP/loxP^* mice) or quasi-Poisson (*wild-type* mice) distribution.

##### FC

The conditioning chamber consisted in an operant chamber 14.0 (w) × 15.9 (l) × 12.7 (h) cm (Med Associates) with steel rods on the floor connected to a scrambler (Med Associates), placed inside a closed ventilated box for sensorial isolation. Mice were introduced into the chamber and light was turned on. After 2-min habituation, three consecutive shocks (0.35 mA, 2-s duration) were applied with an intertrial interval of 90 s. One minute after the third shock, light turned off and mice were removed and returned to their home cages. Twenty-four hours later, mice were reintroduced into the chamber for 5 min to test contextual conditioned freezing. Mice were videotaped during the entire training and testing sessions. Freezing behavior was hand scored by videotape observation and freezing or active avoidance were determined in continuous 4-s bins. Freezing behavior was presented as the percentage of freezing events over total events. Differences in the percentage of time freezing behavior in each stage of the training (baseline, after first shock, after second shock and after third shock) and the testing were evaluated by MANOVA and further validated by univariate tests. Univariate differences in the percentage of time freezing were evaluated by Wald test in a GLM with quasi-binomial distribution for baseline and test stages, and by likelihood-ratio test in a GLMM with quasi-binomial distribution with the Group as between subjects’ factor and Shock as within-subject factor with three levels (shock 1, shock 2, and shock 3) in the case of the postshocks part of the training.

### Software and statistics

All data represent the mean ± SEM and were graphed using GraphPad Prism Software (version 5.01, 2007 GraphPad Software Inc.) and analyzed using R Studio (version 3.2.3). Data with continuous variables were analyzed by Student’s *t* test, ANOVA or repeated measures ANOVA. Normal distribution and homoscedasticity were verified with Shapiro test, QQ-plot and Levene test (library “car”). When appropriate, variance was modeled with VarIdent function (library “nlme”). Discrete data were analyzed with GLMs or GLMM with Poisson error structure (link:loggit). Variables corresponding to rates of success over total trials were analyzed using GLM with binomial error structure (link:log). For GLM and GLMM, significances were evaluated with “likelihood-ratio test” comparing nestled models, or Wald test in the case of quasi-binomial models. Assumptions were evaluated assessing the absence of patterns in Pearson’s residuals graph and calculating the dispersion parameter to assess subdispersion or overdispersion. When assumptions were not achieved, negative-binomial or quasi-Poisson distributions instead Poisson, or quasi-binomial instead binomial, were used. For MANOVA, univariate and multivariate normality (Royston’s test, library “MVN”) and homogeneity of covariance matrices (Box test, library “biotools”) and absence of multivariate outliers (Mahalanobis distance) were verified.

### Code accessibility

Data analysis and tables can be accessed at https://github.com/casey-e/Casey-et-al-2021.

## Results

### Presynaptic and postsynaptic distribution of D2R in the CeA

**Figure 1.**
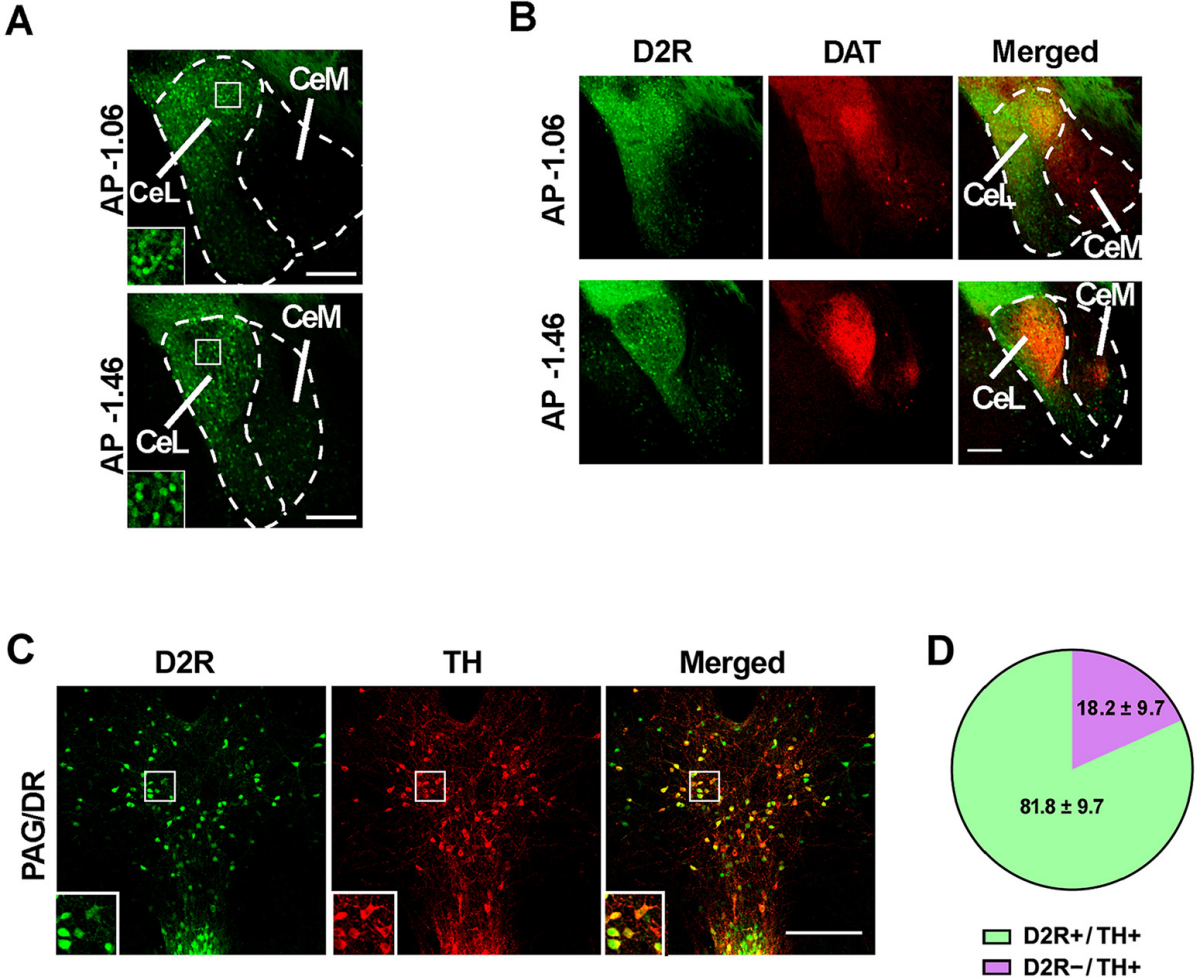
Postsynaptic and presynaptic distribution of D2R-expressing neurons. ***A***, Representative confocal microscopy of coronal sections at two anteroposterior levels of the CeA of a *Drd2*-EGFP mouse. Magnifications of boxed areas are shown in the left bottom corners. ***B***, Representative histology of D2R-expressing neurons (green, left) in CeA coronal sections of *Drd2*-EGFP.*Dat*^+/IRES-Cre^.Ai14 triple transgenic mice. DAergic fibers (reported by Cre-induced tdTomato, in red) and their overlap with D2R-expressing neurons is shown at the center and right, respectively. ***C***, ***D***, Analysis of *Drd2* expression in DAergic neurons of the PAG/DR. ***C***, Representative confocal microscopy of TH immunofluorescence on a PAG/DR coronal section of a *Drd2*-EGFP mouse. Magnifications of boxed areas are shown. ***D***, Percentage of D2R+ and D2R– neurons from total TH+ neurons in the vPAG/DR (mean ± SEM, *n* = 3 mice). CeL, CeA, lateral part; CeM, CeA, medial part; AP, antero-posterior. Scale bars: 200 μm.

To identify neurons expressing D2Rs in the CeA, we performed a comprehensive histologic analysis of coronal brain slices of *Drd2*-EGFP BAC transgenic mice (Gong et al., 2003). In agreement with previous reports using *in situ* hybridization (Kim et al., 2017; McCullough et al., 2018a), we found a wide distribution of neuronal cell bodies expressing D2Rs along the lateral division of the CeA (CeL) and through the entire antero-posterior axis of this division (Fig. 1*A*). In contrast, D2R-positive cell bodies were only sparsely detected in the medial division (CeM), and mainly located near the boundaries of the CeL (Fig. 1*A*). To analyze whether D2R-expressing neurons are innervated by DAergic terminals we crossed *Drd2*-EGFP mice with mutant mice carrying a *Dat^IRES-Cre^ knockin* allele (Bäckman et al., 2006) and the Cre-inducible *tdTomato* reporter gene Ai14 (Madisen et al., 2010) that, together, label DAergic neurons with red fluorescence. Using these triple transgenic mice, we found that D2R-expressing neurons and DAergic fibers largely overlap in the CeL (Fig. 1*B*), suggesting that D2Rs of the CeA are functional receptors regulated by DA. In addition, we detected a low density of DAergic fibers in the CeM, a CeA division with sparse D2R-expressing neurons (Fig. 1*B*).

Then, we investigated whether the major DAergic input to the CeA, originating from the vPAG/DR (Hasue and Shammah-Lagnado, 2002; Li et al., 2016), expresses D2Rs that may function as D2 autoreceptors in the CeA. Using *Drd2*-EGFP mice we found that most DAergic vPAG/DR neurons also express the reporter transgene driven by *Drd2* (81.8 ± 9.7% of total TH-immunoreactive neurons; Fig. 1*C*,*D*), a result that supports prior data suggesting D2 autoreceptor regulation of DA release in the CeA (Bull et al., 1991). Together, these data indicate that D2Rs in the CeA are present both postsynaptically and presynaptically.

### D2Rs stimulate PKCδ+ neurons in the CeA

**Figure 2.**
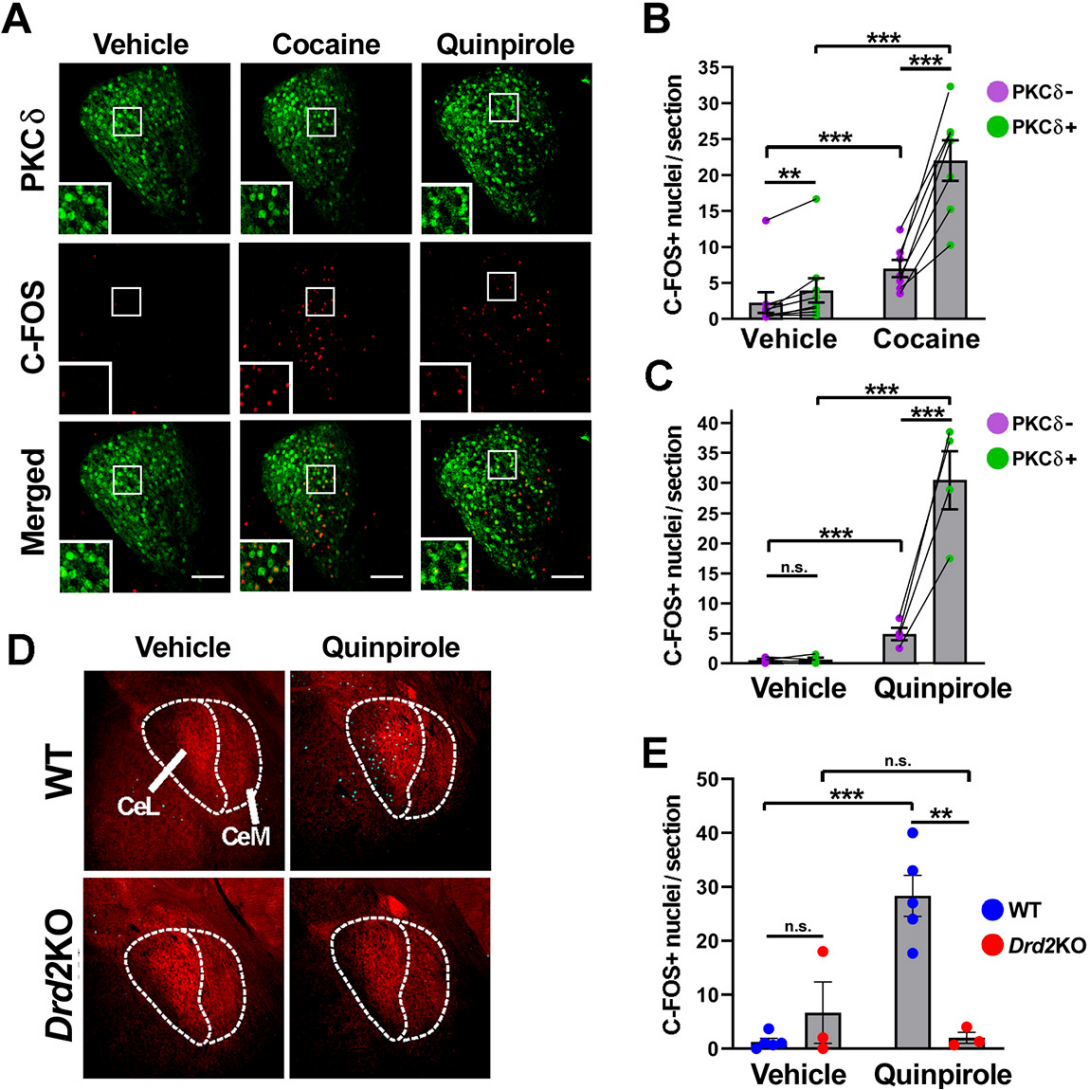
D2R stimulation activates CeA PKCδ neurons. ***A***, Representative histology of PKCδ (green, top) and c-FOS (red, center) double immunofluorescence in coronal sections of the CeA of WT mice receiving vehicle, cocaine (20 mg/kg, i.p.) or quinpirole (1 mg/kg, i.p.). Insets are magnifications of the boxed areas. ***B***, Quantification of c-FOS activation induced by cocaine; lines connect data from the same mouse. Two-way GLMM with negative binomial family (link: log); likelihood-ratio test: type of neuron, x^2^(1) = 66.1, *p* < 0.0001; drug, x^2^(1) = 15.4, *p* < 0.0001; drug × type of neuron, x^2^(1) = 3.5, *p* = 0.06; vehicle, *n* = 9; cocaine, *n* = 7; *post hoc* Tuckey’s test shown. ***C***, Quantification of c-FOS activation induced by quinpirole; lines connect data from the same mouse. Two-way GLMM with Poisson family (link: log); likelihood-ratio test: drug × type of neuron, x^2^(1) = 9.3, *p* = 0.03; vehicle, *n* = 4; quinpirole, *n* = 4; *post hoc* Tuckey’s test shown. ***D***, Representative double immunofluorescence for c-FOS (cyan) and TH (red) in the CeA and (***E***) quantification of c-FOS expression in the CeA induced by saline (Veh) or quinpirole (Quin; 1 mg/kg, i.p.) given to *wild-type* (wt) or *Drd2*KO (KO) mice. Two-way GLMM with negative binomial family (link: log); likelihood-ratio test: drug × genotype, x^2^(1) < 8.1, *p* < 0.01; *post hoc* Tuckey’s test shown. Veh: WT, *n* = 5; KO, *n* = 3; Quin: WT, *n* = 5; KO, *n* = 3. CeL, CeA, lateral part; CeM, CeA, medial part. Scale bars: 100 μm. ***p < 0.001, **p < 0.01, n.s. *p* > 0.05.

The neuronal circuits of the CeA involved in the processing of fear-induced behaviors have been described in detail (Ciocchi et al., 2010; Janak and Tye, 2015; Kim et al., 2017). A group of CeL neurons expressing the molecular marker PKCδ+ is known to reduce the expression of defensive behaviors by inhibiting projection neurons of the CeM (Ciocchi et al., 2010; Haubensak et al., 2010; Tye et al., 2011). Conversely, PKCδ– neurons of the CeL facilitate the expression of fear responses by inhibiting PKCδ+ CeL neurons (Ciocchi et al., 2010; Haubensak et al., 2010). To study the participation of D2Rs in the CeA, we investigated whether PKCδ+ and PKCδ– neurons are differentially activated by D2R stimulation using an immunofluorescence coexpression analysis of PKCδ and the immediate early gene *c-Fos*. We found that both the DA transporter blocker cocaine (20 mg/kg, i.p.) and the D2R agonist quinpirole (1 mg/kg, i.p.) increased the number of c-FOS+ nuclei in the CeL. Furthermore, the increase in c-FOS expression was significantly greater in PKCδ+ neurons than in PKCδ– neurons (likelihood-ratio test for the interaction between drug and cell type), being 3-fold greater in cocaine-injected mice (Fig. 2*A*,*B*) and 6-fold greater in quinpirole-injected mice (Fig. 2*A*,*C*), in agreement with a previous report (De Bundel et al., 2016). The effect of quinpirole was mediated exclusively by D2Rs, since the number of c-FOS+ nuclei was not increased when given to *Drd2*−/− (*knockout*) mice (Fig. 2*D*,*E*). These results indicate that stimulation of D2Rs activate PKCδ+ neurons in the CeA. Because PKCδ+ neurons in the CeA have been shown to mediate anxiolytic effects (Haubensak et al., 2010; Cai et al., 2014), we hypothesized that D2Rs present in this region regulate risk assessment.

### Ablation of postsynaptic D2Rs in the CeA increases avoidance in exploratory tasks

**Figure 3.**
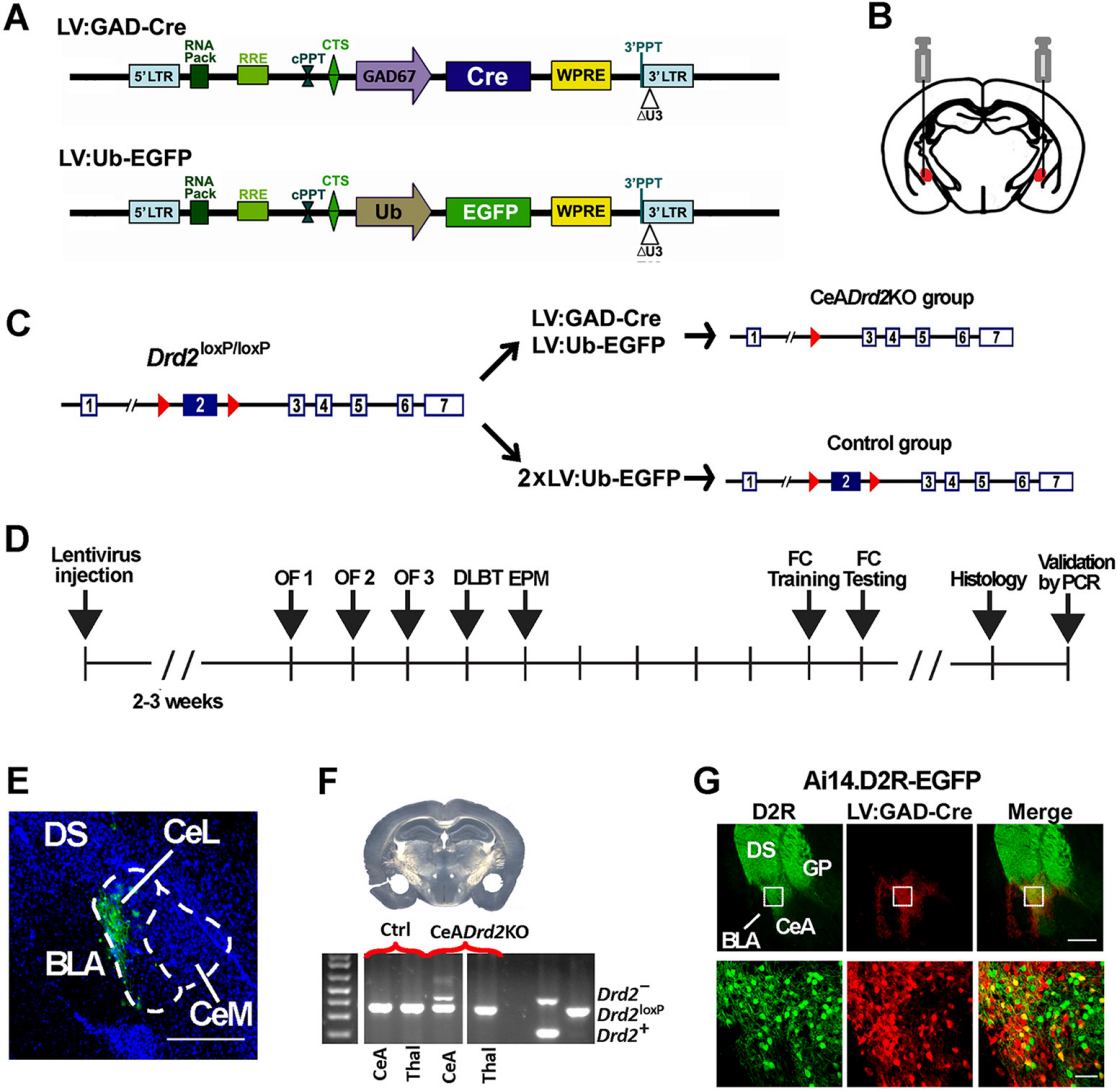
Strategy for selective genetic ablation of D2Rs in the CeA. ***A***, Schematic of the lentiviral vectors used to express Cre driven by the GAD67 promoter and EGFP driven by the ubiquitin promoter. ***B***, Schematic of lentiviral vectors bilateral injections. ***C***, Strategy for generating CeA*Drd2*KO and control mice. ***D***, Experimental timeline. OF, exploratory activity in an open field; EPM, elevated plus maze; DLBT, dark/light box test; FC, fear conditioning. Each vertical bar indicates 1 day. ***E***, Representative histology of a coronal section showing co-injections of LV:GAD-Cre and LV:Ub-EGFP into the CeA. ***F***, LV:GAD-Cre-induced recombination in *Drd2*^loxP/loxP^ mice was verified by PCR with primers detecting deleted (*Drd2*^–^), floxed (*Drd2^loxP^*), and *wild-type* (*Drd2*^+^) alleles from biopsies containing the CeA or thalamus, as negative control, of CeA*Drd2*KO (*n* = 4) and control (*n* = 4) mice. Result of a mouse per group are shown, followed by a negative control (water) and two positive controls**. *G***, Representative histology of a coronal brain section of a *Drd2*-EGFP.Ai14 double transgenic mouse receiving a stereotaxic injection of LV:GAD-Cre into the CeA. D2R+ (green), Cre-induced tomato (red).

To investigate the participation of amygdalar D2Rs in behavioral reactions involved in risk assessment we generated mice partially lacking postsynaptic D2Rs in the CeA and tested them in several approach/avoidance conflict paradigms. The molecular strategy used to specifically ablate postsynaptic D2Rs without affecting D2 autoreceptors was based on the expression of Cre recombinase directly into the CeA of *Drd2^loxP/loxP^* mice, a homozygous strain carrying conditional *Drd2* null alleles (Bello et al., 2011). To this end, we used a lentiviral vector expressing the Cre recombinase under the control of the GAD67 promoter (Tolu et al., 2010; LV:GAD-Cre; Fig. 3*A*, top) together with a lentiviral vector expressing EGFP under the control of the human ubiquitin promoter that allows later visualization of the injection site (LV:Ub-EGFP; Fig. 3*A*, bottom). The use of the GAD67 promoter to drive Cre was based on the fact that CeA neurons are GABAergic (McDonald, 1982; Ehrlich et al., 2009). We performed bilateral stereotaxic co-injections of LV:GAD-Cre and LV:Ub-EGFP into the CeA of *Drd2^loxP/loxP^* mice (from now on, CeA*Drd2*KO mice; Fig. 3*B*), to excise the essential exon 2 of *Drd2* (Fig. 3*C*). Control *Drd2^loxP/loxP^* mice received LV:Ub-EGFP injections only (Fig. 3*C*). Two to three weeks after this procedure, mice were subjected to a battery of approach/avoidance conflict paradigms where their behaviors were evaluated (Fig. 3*D*). At the conclusion of these tests, the injection sites were determined histologically for each mouse (Fig. 3*E*). Cre-induced deletion of exon 2 from the *floxed Drd2* alleles was verified by PCR using DNA extracted from the CeA (Fig. 3*F*). In addition, to validate the efficacy of the viral vectors to target CeA neurons expressing D2Rs, we injected LV:GAD-Cre into the CeA of compound *Drd2*-EGFP.Ai14 transgenic mice and confirmed that the injections were limited to the CeA inducing *tdTomato* expression in 38 ± 14% (average ± confidence interval, *n* = 5 injections) of *Drd2*-EGFP+ neurons at the injection site (Fig. 3*G*). Noteworthy, we did not detect tdTomato+ neurons in any of the input areas projecting to the CeA, demonstrating that transduction of the LV was restricted to postsynaptic neurons and was not retrogradely transported.

**Figure 4.**
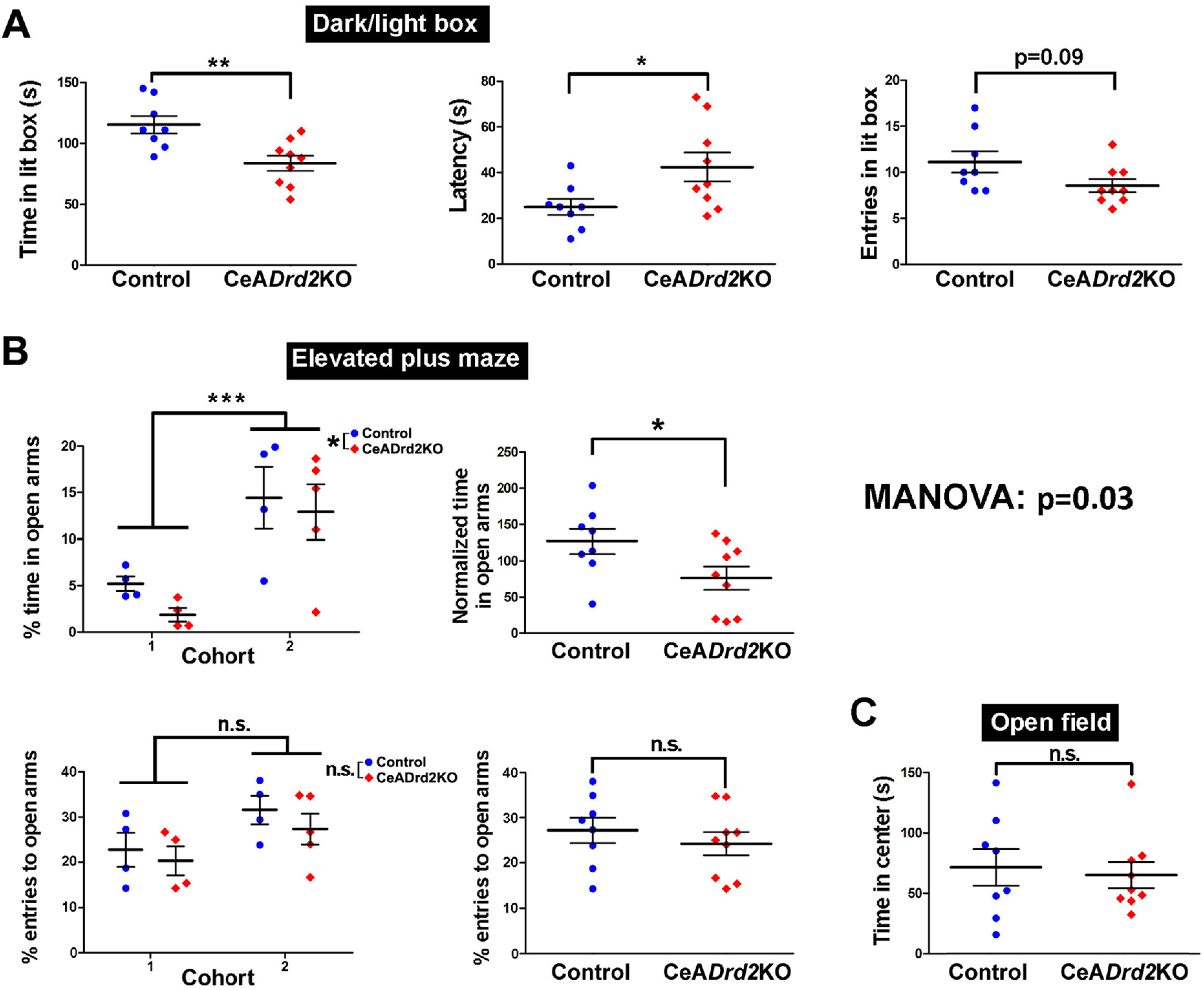
D2R ablation from the CeA increases avoidance in exploratory tasks. The result of a MANOVA including every measure is shown at the center, right. ***A***, Dark/light box. Left, Time spent in the lit chamber. Student’s *t* test, *t*_(15)_ =3.4; *p* = 0.004. Middle, Latency to first entry into lit chamber in a dark/light box test. Student’s *t* test; *t*_(15)_ = −2.3; *p* = 0.034. Right, Number of entries into the lit chamber in a dark/light box test. GLM with Poisson family (link: log); Wald test, z = −1.7, *p* = 0.092. ***B***, EPM. Top, Percentage of time on open arms. Only in this measure, the cohort of mice had a significant effect. Therefore, the percentage of time on open arms was analyzed in two different ways (see the Materials and Methods, EPM). Top-left, Percentage of time in open arms, two-way (group × cohort) ANOVA with variance modeling (“VarIdent” function, applied to cohort factor), group, *F*_(1,13)_ = 8.9, *p* = 0.011; Cohort, *F*_(1,13)_ = 19.9, *p* < 0.001; group × cohort, *F*_(1,13)_ = 0.16, *p* = 0.69. Top-right, Percentage of time in open arms normalized to the cohort average. Student’s *t* test; *t*_(15)_ = 2.1; *p* = 0.049). Bottom, Percentage of entries to open arms over total entries (open+closed). Since statistical differences between cohorts were not detected, normalization to cohort averages was not performed. Bottom-left, Percentage of entries to open arms over entries to open and closed arms in an EPM, with data separated according to the cohort. Two-way GLMM with quasi-binomial family (link: logit); Wald test; group, *t* = −0.31, *p* = 0.76; cohort, *t* = 1.86, *p* = 0.09; group × cohort, *t* = 0.1, p 0.92. Bottom-right, Percentage of entries to open arms, with data of both cohorts pulled. GLMM with quasi-binomial family (link: logit); Wald test, *t* = −0.37, *p* = 0.72. ***C***, Open field, Time in center of the arena during the first 5 min of exposure. Student’s *t* test, *t*_(15)_ = 0.35, *p* = 0.73. CeA*Drd2*KO, *n* = 9; Ctrl, *n* = 8 in all experiments. ***p < 0.001, **p < 0.01, *p < 0.05, n.s. *p* > 0.05.

A MANOVA for measures of approach/avoidance conflicts demonstrated that CeA*Drd2*KO mice are significantly different from control mice (one-way MANOVA, Pillai = 0.7, approximated *F*_(1,15)_ = 3.9, *p* = 0.03; Fig. 4). In agreement, using univariate analysis we found that CeA*Drd2*KO mice spent significantly less time than control mice in the lit compartment of the dark/light box (Fig. 4*A*, left), showed greater latencies to enter for the first time into this compartment (Fig. 4*A*, middle), and entered fewer times into the lit box [marginal difference (*p* = 0.09); Fig. 4*A*, right]. Similarly, CeA*Drd2*KO mice spent significantly less time on the open arms of the EPM than their control siblings (Fig. 4*B*, top-left and right), and the percentage of entries into the open arms over the total number of arm entries followed the same trend (Fig. 4*B*, bottom-left and right). Differently, the time spent in the center of the OF was similar in CeA*Drd2*KO and control mice (Fig. 4*C*).

**Figure 5.**
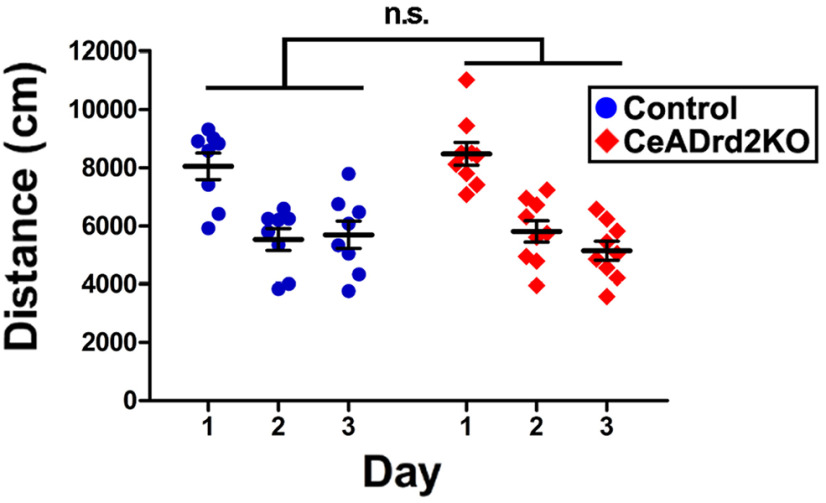
D2R ablation from the CeA does not affect locomotion. Daily distance traveled by CeA*Drd2*KO and control mice during 30-min sessions of open field exploration; two-way ANOVA with repeated measures; group, *F*_(1,45)_ = 0.001, *p* > 0.1; days, *F*_(2,45)_ = 76.3, *p* < 0.001; group × days, *F*_(2,45)_ = 2, *p* > 0.1. CeADrd2KO, *n* = 9 and Ctrl, *n* = 8. n.s., *p* > 0.05.

The increased avoidance behaviors observed in CeA*Drd2*KO mice cannot be attributed to lower motivation for exploring new environments or to lower locomotor activity, because LV:GAD-Cre injections did not affect the distance traveled in the OF during the first day (when the arena is a novel environment) or in the second or third day (when the arena is a known environment; Fig. 5).

**Figure 6.**
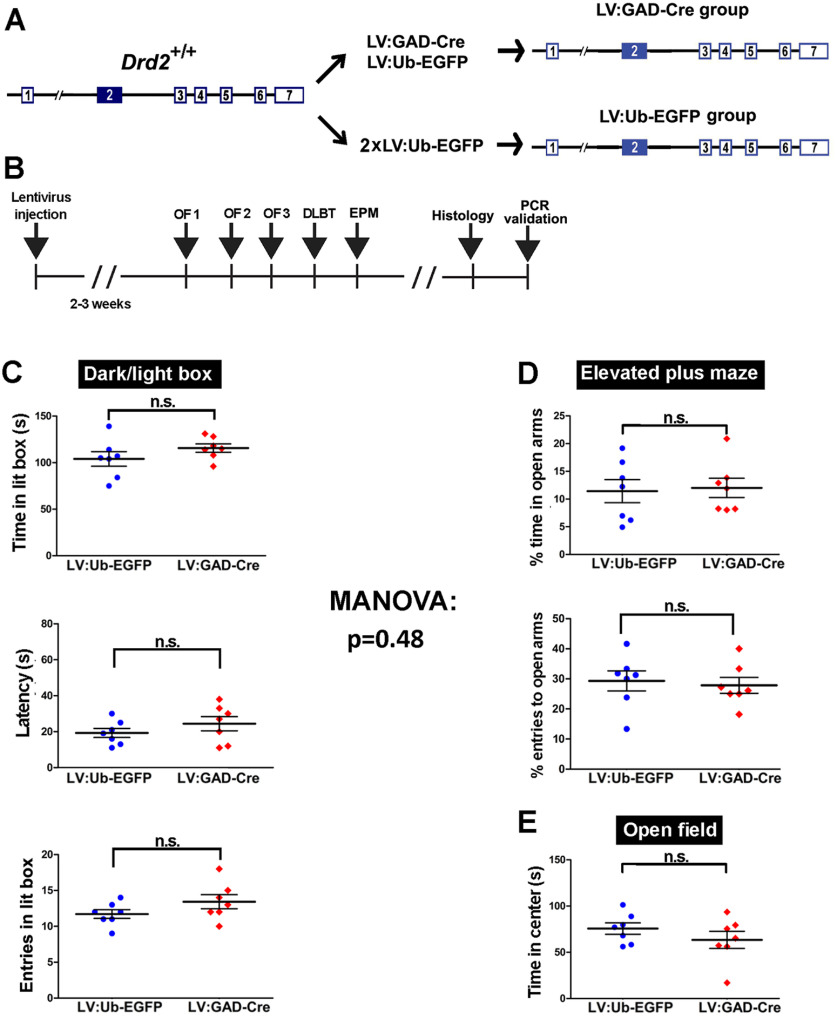
LV:GAD-Cre injections into the CeA of *Drd2*^+/+^ mice does not alter behaviors in exploratory tasks. ***A***, Experimental design with viral injections. ***B***, Experimental timeline. OF, exploratory activity in an open field; EPM, elevated plus maze; DLBT, dark/light ox test. Each vertical bar indicates 1 day. ***C–E***, Avoidance behavior in exploratory tasks. The result of a MANOVA including every measure is shown in the center. ***C***, Dark/light box. Top, Time in the lit chamber. Student’s *t* test, *t*_(12)_ = 1.3; *p* = 0.22. Middle, Latency to first entry to light chamber; Student’s *t* test; *t*_(12)_ = 1.1, *p* = 0.29. Bottom, Number of entries to light chamber; GLM with quasi-Poisson family (link: log); Wald test, z = 1.51, *p* = 0.16. ***D***, EPM. Top, Percentage of time on the open arms over the time on open and closed arms; Student’s *t* test; *t*_(12)_ = 0.21; *p* = 0.83. Bottom, Percentage of entries to open arms over total. GLMM with quasi-binomial family (link: logit); Wald test, z = −0.49, *p* = 0.64. ***E***, Open field, Time in center of the arena during the first 5 min of exposure; Student’s *t* test, *t*_(12)_ = 1.1, *p* = 0.29. LV:GAD-Cre, *n* = 7; LV:Ub-EGFP, *n* = 7 in all the experiments. **p < 0.01, *p < 0.05, n.s. *p* > 0.05.

**Figure 7.**
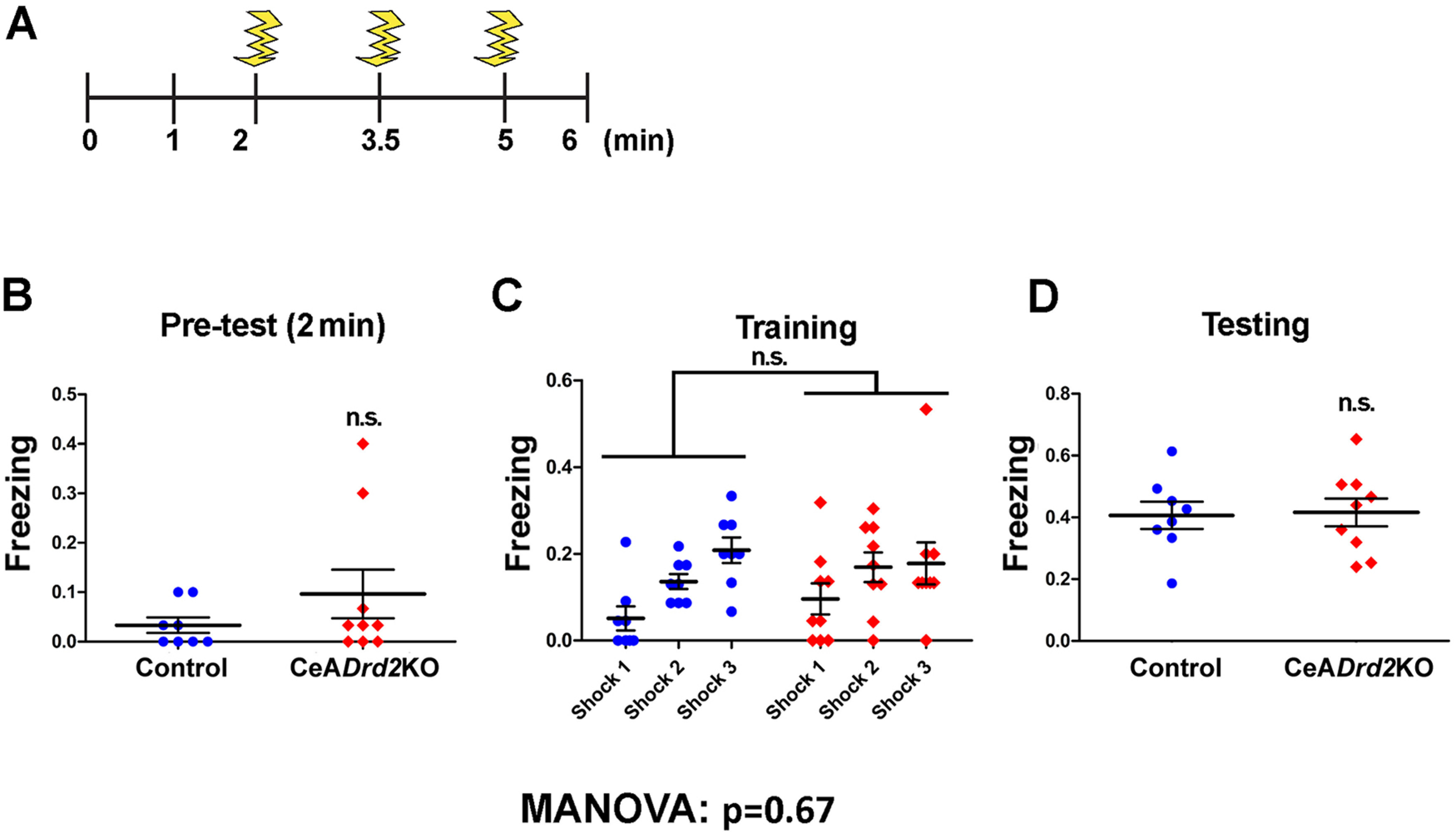
Mice lacking D2R in the CeA express unaltered fear-conditioning memory. ***A***, Contextual FC training protocol. ***B***, Percentage of freezing during the first 2 min of chamber habituation during the training [pre-test, GLM with quasi-binomial family (link: log); Wald test, z = 1.23, *p* = 0.23]. ***C***, Percentage of freezing after each foot shock [training session, GLMM with quasi-binomial family (link: log); LRT test; group × shock, X^2^(1) = 2.8, *p* = 0.24; group, X^2^(1) = 0.2, *p* = 0.65; shock, X^2^(1) = 21.7, *p* < 0.001]. ***D***, Percentage of freezing during re-exposition to the conditioning chamber, 24 h after the training [testing session, GLM with quasi-binomial family (link: log); Wald test, z = 0.15, *p* = 0.88]. Result of MANOVA including all variables is shown. CeADrd2KO, *n* = 9 and Ctrl, *n* = 8, n.s. *p* > 0.05.

To verify that the increased avoidance behaviors described in Figure 4 were caused by the selective elimination of postsynaptic D2Rs from the CeA, we administered LV:GAD-Cre and LV:Ub-EGFP directly into the CeA of *Drd2*^+/+^ mice (*wild-type* mice without loxP sites flanking exon 2; Fig. 6*A*) and challenged them with the same battery of behavioral tests (Fig. 6*B*). *Drd2^+/+^* mice receiving LV:GAD-Cre injections did not show any behavioral difference in these tests compared with mice receiving LV:Ub-EGFP only (one-way MANOVA, Pillai = 0.5, approximated *F*_(1,12)_ = 1.0, *p* = 0.48; Fig. 6*C–E*), indicating that the enhanced avoidance displayed by CeA*Drd2*KO mice was in fact because of the elimination of postsynaptic D2Rs from the CeA. Finally, CeADrd2KO mice did not differ from control mice in the time spent freezing when re-exposed to a chamber previously associated to unavoidable foot-shocks (one-way MANOVA, Pillai = 0.2, approximately *F*_(1,15)_ = 0.6, *p* = 0.67; Fig. 7*A–D*). Together, these results indicate that partial ablation of postsynaptic D2R in the CeA increases avoidance in exploratory tasks and suggest that amygdalar D2Rs control behavioral responses to riskier environments.

## Discussion

In this study, we used a conditional site-specific genetic approach in mice and found that partial ablation of postsynaptic D2Rs in the CeA increased avoidance behaviors in approach/avoidance conflict paradigms, suggesting that DA stimulation of amygdalar D2Rs regulates behavioral reactions to potential threats present in novel environments. The enhanced avoidance behavior observed in mice partially lacking D2Rs in the CeA was more profound when mice were studied in the DLBT than in the EPM. This milder, although significant, effect was likely caused by a strong and significant Cohort effect in the time spent on the open arms of the EPM, probably driven by variable and undetermined environmental conditions existing at the different moments when the two mouse cohorts were studied. Noteworthy, a two-way ANOVA indicated significant main effects of the group and the cohort, indicating that the effect of D2R ablation in the CeA had similar effects in both cohorts.

Although D2Rs in the CeA have been previously studied using pharmacological compounds (Guarraci et al., 2000; De la Mora et al., 2012; De Bundel et al., 2016), we believe this is the first report investigating the effects on defensive behaviors elicited by the genetic ablation of postsynaptic D2Rs directly into the CeA. Although the lentiviral-mediated approach used in this study induced only a partial ablation of D2Rs in the injected CeA (Fig. 3*G*), this limited deletion showed to be enough to alter the behavioral reactions of potentially dangerous environments (Fig. 4), without affecting spontaneous locomotor activity (Fig. 5) or fear memory (Fig. 7). In contrast, previous studies based on local applications of D2R antagonists into the CeA had shown no changes in risk avoidance behavior, although elicited impaired reactions to FC (Guarraci et al., 2000) and unconditioned fear (De la Mora et al., 2012). Such differences may be because of the fact that pharmacological agents not only act on postsynaptic D2Rs but also on D2 autoreceptors that control DA release (Bello et al., 2011). Indeed, using *Drd2*-EGFP mice, we found that most TH-immunoreactive neurons present in the vPAG/DR also express *Drd2* (Fig. 1*C*,*D*), a result supported by data collected in the Allen Mouse Brain Atlas showing intense labeling of *Drd2* mRNA in vPAG/DR cell bodies (Lein et al., 2007). Furthermore, the D2R antagonist sulpiride has been shown to potentiate DA release elicited by optogenetic activation of vPAG/DR terminals reaching the bed nucleus of the stria terminalis (Yu et al., 2021), whereas an *in vitro* study demonstrated that D2 autoreceptors regulate DA release in the CeA (Bull et al., 1991). Thus, functional evidence (Bull et al., 1991; Yu et al., 2021), together with histologic results shown in this work and in Lein et al. (2007), indicates that DA release in the CeA is regulated by presynaptic D2 autoreceptors. Consequently, pharmacological activation or blockade of D2Rs present in the CeL are likely to alter DA release and affect the activity of CeA neurons expressing postsynaptic DA receptors.

In agreement with a role of D2Rs of the CeA in risk avoidance, our pharmacological results show D2R-mediated activation of CeL PKCδ+ cells (Fig. 2), a group of neurons that has been shown to elicit anxiolytic-like effects in approach/avoidance conflict tests (Cai et al., 2014; Griessner et al., 2021). Considering that D2Rs are generally Gi/o-coupled receptors that decrease neuronal excitability (Innis and Aghajanian, 1987; Lacey et al., 1987; Uchida et al., 2000), the excitatory effect on PKCδ+ neurons is likely because of polysynaptic transmission, a mechanism that needs to be further clarified in future studies. Because DA agonists were applied systemically in this study, we cannot rule out the possibility that the D2R-mediated activation of CeL PKCδ+ cells was indirectly driven by stimulation of D2Rs located in other brain areas. Although specific subpopulations of PKCδ+ neurons drive defensive behaviors (Cui et al., 2017; Kim et al., 2018), the PKCδ+ neurons activated by quinpirole and cocaine most likely reduce anxiety-like behaviors, as c-FOS expression in the CeA elicited by cocaine or amphetamine can be prevented by exposure to stressful environments (Day et al., 2001, 2005, 2008), suggesting that indirect DA agonists and potential threats drive opposite effects in CeA microcircuits. Our demonstration that partial ablation of D2Rs from the CeA increased avoidance in exploratory tasks (Fig. 4) without impairing locomotor activity (Fig. 5), together with the finding that D2Rs activate PKCδ+ neurons in the CeL (Fig. 2) suggest that amygdalar D2Rs are potential therapeutic targets for anxiolytic compounds. Interestingly, the D2/D3 agonist ropinirole has shown anxiolytic effects together with motor improvements in Parkinson’s disease patients (Rogers et al., 2000; Rektorova et al., 2008; Mavrikaki et al., 2014). In addition, it has been recently shown that CeL PKCδ+ neurons are necessary and sufficient for the anxiolytic effect of benzodiazepines (Griessner et al., 2021).

In summary, here, we demonstrate that removing D2Rs in the CeA increases anxiety-like behaviors, as evidenced by reduced exploration of riskier environments. This result is consistent with the work of De Bundel and colleagues, in which blocking D2Rs in the CeA after a FC test leads to fear generalization, a clinical marker of anxiety disorders (De Bundel et al., 2016). In contrast, D1R stimulation in the CeA facilitated fear learning (Guarraci et al., 1999; Groessl et al., 2018) and expression (Guarraci et al., 1999), without affecting anxiety-like behaviors (Groessl et al., 2018). Thus, we hypothesize that DA in the CeA reduces anxiety-like behaviors via D2Rs and enhances fear learning and memory via D1R. A similar mechanism has been proposed for DA acting in the striatum, where DA regulates behavioral selection by enhancing the contrast between stronger and weaker inputs reaching striatal neurons (for review see, Nicola et al., 2004). We propose that in the CeA, DA strengthens the value of aversive stimuli via D1Rs (Guarraci et al., 1999; Groessl et al., 2018) and attenuates signals from low risk or neutral stimuli via a D2R-based mechanism, as we found here and in agreement with a previous report (De Bundel et al., 2016). Further studies will be necessary to address the participation of other DAergic receptors in amygdalar circuits mediating responses to fearful, dangerous, and high-risk situations.

## Synthesis

Reviewing Editor: Michael Michaelides, NIDA-NIH Decisions are customarily a result of the Reviewing Editor and the peer reviewers coming together and discussing their recommendations until a consensus is reached. When revisions are invited, a fact-based synthesis statement explaining their decision and outlining what is needed to prepare a revision will be listed below. The following reviewer(s) agreed to reveal their identity: Miriam Bocarsly.

Reviewer 1

In this manuscript Casey et al. investigate the role of D2R in the central nucleus of the amygdala, and their role in defensive behaviors. D2R stimulation enhanced Fos activity in PKCδ neurons suggesting an increase in their activity. Deletion of post-synaptic D2Rs in the CeA enhanced risk avoidance in a battery of approach avoidance tasks, EPM and LD box especially. The study is interesting and builds on work examining the role of D2 circuits, and site directed pharmacology. The writing is clear and easy to follow.

I have a few concerns that are relatively minor in nature outlined below:

Introduction fails to mention the Kim et al paper (https://www.pnas.org/content/115/45/E10730) that examines the role of a D2 containing circuit from the CeA to the BNST in the manifestation of risk behaviors.

The case for presynaptic D2 receptors as written in the results is a bit weak. The authors could cite Yu et al., Neuron, 2021 for evidence that PAG/A10 DA neurons show altered release of DA when challenged with a D2 antagonist.

The D2 knockout mice mentioned in figure 2 are not mentioned in the animals in the methods section.

In figure 2 it is a bit confusing why there are lines connecting the PKCδ- and PKCδ+ cells? Are these cells supposed to be from the same animal and is that why they are grouped this way for comparison? Is this why the MANOVA is run?

Can the authors explain why they observed such different behavior between the cohorts in the EPM? This seems to not be addressed and is also again maybe why a MANOVA was used due to unequal variance?

Reviewer 2

Casey et al., implement creative methods to begin to explore specific subpopulations of dopamine D2 receptors in the central amygdala. The authors conclude that postsynaptic D2Rs in the CeL modulate risk avoidance in exploratory tasks. As a D2R enthusiast, I think that the overall idea presented, namely defining, and delineating the roles of specific subpopulations of D2Rs in behavior, is important. I do have some concerns in methods and conclusions that cause hesitation.

1. This study relies heavily on the D2R-EGFP mouse, which has been shown to have up to 40% increase in membrane expression of D2Rs and twofold increase in D2R mRNA levels in the striatum compared to wild-type mice. While I am unaware of a similar analysis in the central amygdala, this is likely the case. Minimally, this potential caveat should be addressed.

2. I am interested in the details of the quinpirole experiment. The drug dose used is incredibly high and known to have profound behavioral effects (immobility for more than an hour, followed by hyperlocomotion). Why was this dose selected? Is this based on previous publications? Further, in this experiment it was concluded that stimulation of the D2Rs activate PKCδ+ neurons in the CeA. While this statement is correct, it should be emphasized that because the drugs were given peripherally, you can’t conclude that it’s the CeA D2Rs leading to increased cFOS in PKCδ+ cells. This connection is suggested and is a valid hypothesis, but beyond the scope of these experiments. It would be interesting to see a study where modulating expression of CeA D2Rs effects activity of PKCδ+ neurons.

3. As the authors note, the data differences on the EPM across cohorts in Figure 4 is puzzling. Collapsing across all the EPM data in this paper, it does seem that cohort 1 is the outlier here. Cohort 1 also seems to be fully driving the statistical difference... which makes me hesitant to draw much of a conclusion from this data. That said, the only clear effect is the difference in the light-dark box. This makes the conclusion that “these results indicate that partial ablation of the postsynaptic D2R in the CeA increases avoidance in exploratory tasks and suggests that amygdalar D2Rs control behavioral responses to riskier environments...” seem to be an overinterpretation of the data. Is it possible that the behavior could reflect variability in the percentage of D2R knockdown, which the authors might be able to analyze in the current data sets? Further, it would be nice to see a summary of the knockdown across mice, rather than just one representative image (fig 3F).

4. More information should be given on specific mice used. Were the Drd2-/- mice full knockouts and where were they obtained? For all mouse strains, were they purchased or bred in house, etc.?

5. The manuscript states mice of both sexes were used-were any sex-specific differences noted?
